# Draft genome sequence of *Paenibacillus* sp. EZ-K15 isolated from wastewater systems

**DOI:** 10.1186/s13104-017-3069-8

**Published:** 2017-12-12

**Authors:** Waleed S. Mohammed, Elvira E. Ziganshina, Elena I. Shagimardanova, Natalia E. Gogoleva, Ayrat M. Ziganshin

**Affiliations:** 1 Department of Microbiology, Institute of Fundamental Medicine and Biology, Kazan (Volga Region) Federal University, Kremlyovskaya str. 18, Kazan, 420008 Russia; 2 Department of Biotechnology, Faculty of Agriculture, Al-Azhar University, Cairo, 11651 Egypt; 3 Laboratory of Extreme Biology, Institute of Fundamental Medicine and Biology, Kazan (Volga Region) Federal University, Kazan, 420021 Russia

**Keywords:** *Paenibacillus* sp., Genome sequencing, Draft genome assembly and annotation, Wastewater

## Abstract

**Objectives:**

*Paenibacillus* species, belonging to the family *Paenibacillaceae*, are able to survive for long periods under adverse environmental conditions. Several *Paenibacillus* species produce antimicrobial compounds and are capable of biodegradation of various contaminants; therefore, more investigations at the genomic level are necessary to improve our understanding of their ecology, genetics, as well as potential biotechnological applications.

**Data description:**

In the present study, we describe the draft genome sequence of *Paenibacillus* sp. EZ-K15 that was isolated from nitrocellulose-contaminated wastewater samples. The genome comprises 7,258,662 bp, with a G+C content of 48.6%. This whole genome shotgun project has been deposited at DDBJ/ENA/GenBank under the accession PDHM00000000. Data demonstrated here can be used by other researchers working or studying in the field of whole genome analysis and application of *Paenibacillus* species in biotechnological processes.

## Objective

*Paenibacillus* species, belonging to the family *Paenibacillaceae*, are rod-shaped Gram-positive or Gram-variable endospore forming aerobic or facultatively anaerobic bacteria, which are able to survive for long periods under adverse environmental conditions. Bacteria belonging to the genus *Paenibacillus* can be isolated from a wide range of environments including humans, animals, plants and the environment [1, 2]. Many species of *Paenibacillus* genus synthesize antimicrobial compounds that can be used as pesticides as well as in medicine, and many species produce enzymes important in bioremediation related technologies. *Paenibacillus* strains can be successfully applied for contaminants removal from a variety of wastewater systems. Also, several *Paenibacillus* strains can be involved in hydrolysis of cellulose and hemicellulose, lignin depolymerization and degradation of various textile dyes, polyvinyl alcohol, diesel fuel, bitumen, polycyclic aromatic hydrocarbons, benzene and other compounds [2]. Hence, more studies at the genomic level are important to clarify our understanding of their ecology, genetics, as well as potential biotechnological applications. Thus, *Paenibacillus* sp. EZ-K15 was isolated from nitrocellulose-contaminated wastewater systems, Kazan, Republic of Tatarstan, Russia [3]. These industrial wastes produce high levels of wastewaters polluted with multifarious dissolved chemical compounds and nitrocellulose particles. Therefore, isolation of bacteria which are able to transform various adverse pollutants and their genome analysis are of high importance for the creation of effective bioremediation strategies [4, 5].

## Data description

*Paenibacillus* sp. EZ-K15 was isolated from nitrocellulose-contaminated wastewater environments, Kazan, Republic of Tatarstan, Russia [3]. The bacterium optimally grown on Luria agar at + 30 °C had been cultivated for 1–2 days. Genomic DNA of the bacterial strain EZ-K15 was extracted using a FastDNA spin kit (#116540600; MP Biomedicals) and a Super FastPrep-1 homogenizer (#116011500; MP Biomedicals) as detailed in the manufacturer’s protocol. Concentration and purity (A260/A280) of the obtained bacterial genomic DNA were measured using a NanoDrop 2000 spectrophotometer (#ND-2000; Thermo Fisher Scientific) and then stored at − 20 °C. The bacterial strain *Paenibacillus* sp. EZ-K15 was morphologically identified and then confirmed by PCR amplification applying universal primers UniBac27f, Bakt_805R and Univ1492r, followed by sequencing using an ABI PRISM 3130xl Genetic Analyzer (#4359571; Applied Biosystems) and phylogenetic analysis (16S rRNA gene sequence, 1437 bp, BLAST identity of 99% to *Paenibacillus lautus*; Table 1). In order to perform whole genome analysis, DNA was fragmented using a Q800R2 Sonicator (#Q800R2-110; Qsonica), and DNA library was then prepared using a NEBNext Ultra DNA Library Prep Kit for Illumina (#E7370S; New England Biolabs) according to the manufacturers’ protocols. Both efficiency of DNA fragmentation and DNA library preparation were monitored using a 2100 Bioanalyzer (#G2939BA; Agilent) and a High Sensitivity DNA kit (#5067-4626; Agilent). Further sequencing was performed using a high-throughput Illumina MiSeq platform (#SY-410-1003; Illumina) at Joint KFU-Riken Laboratory, Kazan Federal University (Kazan, Russia) by a MiSeq Reagent Kit v2PE 500 cycles (#MS-102-2003; Illumina). Sequence reads quality was assessed using PRINSEQ lite version 0.20.4 [6], the genome was assembled using Velvet version 1.2.10 [7], and the ordering of contigs was achieved using Mauve version 2.4.0 [8]. The whole genome sequence of *Paenibacillus* sp. EZ-K15 was annotated using the Rapid Annotation System Technology server [9]. The rRNA and tRNA genes were identified using RNAmmer 1.2 [10] and tRNA scan-SE 1.23 [11], respectively. Comparison of the genomic feature of *Paenibacillus* sp. EZ-K15 with several *Paenibacillus* strains was performed using data obtained from EzBioCloud database [12].

**Table 1:** Overview of data files/data sets

Label	Name of data file/data set	File types (file extension)	Data repository and identifier (DOI or accession number)	License
Data file 1	Whole genome shotgun project	FASTA	DDBJ/ENA/GenBank (accession PDHM00000000)	CC-BY
Data file 2	16S rRNA gene sequence	FASTA	GenBank (accession MG050738)	CC-BY

The draft genome sequence of *Paenibacillus* sp. EZ-K15 composed of 36 contigs ranging from 512 to 911,265 bp with a total size of 7,258,662 bp, a G+C content of 48.6% and N50 of 242,001 bp. The Rapid Annotation System Technology server predicted 6682 coding sequences where 2551 coding sequences (39%) were annotated as seed subsystem features and 4131 coding sequences (61%) were annotated as outside of the seed subsystem. In total 4560 and 2122 coding sequences were assigned as non-hypothetical and hypothetical, accordingly. The genome was shown to encode at least 3 rRNAs and 66 tRNAs. The strain *Paenibacillus* sp. EZ-K15 possesses a substantial number of genes responsible for denitrification and nitrate/nitrite ammonification (e.g., for nitrate released during nitrocellulose denitration) as well as for metabolism of aromatic compounds, including genes involved in benzoate, gentisate and some other compounds biodegradation. Numerous genes responsible for resistance to toxic compounds, including arsenic, mercury, cadmium, as well as chromium compounds, were additionally detected. This resistant strain may have future usefulness in bioremediation of various sites.

## Limitations

Current data is based on the draft level genome sequence, due to which exact length of the genome, synteny, number of rRNA and repetitive elements cannot be reported. In addition, whether the genome consists of any plasmid/s or extra-chromosomal DNA cannot be certainly predicted.
